# Virulence factors and antibiograms of *Escherichia coli* isolated from diarrheic calves of Egyptian cattle and water buffaloes

**DOI:** 10.1371/journal.pone.0232890

**Published:** 2020-05-11

**Authors:** Nader M. Sobhy, Sarah G. A. Yousef, Hamada A. Aboubakr, Muhammad Nisar, Kakambi V. Nagaraja, Sunil K. Mor, Robert J. Valeris-Chacin, Sagar M. Goyal

**Affiliations:** 1 Department of Animal Medicine, Faculty of Veterinary Medicine, Zagazig University, Zagazig, Sharkia, Egypt; 2 Department of Veterinary Population Medicine, University of Minnesota, St. Paul, Minnesota, United States of America; 3 Food Science and Technology Department, Faculty of Agriculture, Alexandria University, El-Shatby, Alexandria, Egypt; 4 Department of Veterinary and Biomedical Sciences, University of Minnesota, St. Paul, Minnesota, United States of America; 5 Department of Animal Health and Public Health, Faculty of Veterinary Sciences, University of Zulia, Maracaibo, Venezuela; Michigan State University, UNITED STATES

## Abstract

Diarrhea caused by *Escherichia coli* in calves is an important problem in terms of survivability, productivity and treatment costs. In this study, 88 of 150 diarrheic animals tested positive for *E*. *coli*. Of these, 54 samples had mixed infection with other bacterial and/or parasitic agents. There are several diarrheagenic *E*. *coli* pathotypes including enteropathogenic *E*. *coli* (EPEC), Shiga-toxin producing *E*. *coli* (STEC), enterotoxigenic *E*. *coli* (ETEC) and necrotoxigenic *E*. *coli* (NTEC). Molecular detection of virulence factors Stx2, Cdt3, Eae, CNF2, F5, Hly, Stx1, and ST revealed their presence at 39.7, 27.2, 19.3, 15.9, 13.6, 9.0, 3.4, and 3.4 percent, respectively. As many as 13.6% of the isolates lacked virulence genes and none of the isolate had LT or CNF1 toxin gene. The odds of isolating ETEC from male calves was 3.6 times (95% CI: 1.1, 12.4; P value = 0.042) that of female calves, whereas the odds of isolating NTEC from male calves was 72.9% lower (95% CI: 91.3% lower, 15.7% lower; P value = 0.024) than that in females. The odds of isolating STEC in winter was 3.3 times (95% CI: 1.1, 10.3; P value = 0.037) that of spring. Antibiograms showed 48 (54.5%) of the isolates to be multi-drug resistant. The percent resistance to tetracycline, streptomycin, ampicillin, and trimethoprim-sulfamethoxazole was 79.5, 67.0, 54.5, and 43.0, respectively. Ceftazidime (14.8%), amoxicillin-clavulanic acid (13.6%) and aztreonam (11.3%) showed the lowest resistance, and none of the isolates was resistant to imipenem. The results of this study can help improve our understanding of the epidemiological aspects of *E*. *coli* infection and to devise strategies for protection against it. The prevalence of *E*. *coli* pathotypes can help potential buyers of calves to avoid infected premises. The antibiograms in this study emphasizes the risks associated with the random use of antibiotics.

## Introduction

Cattle and water buffaloes (*Bubalus bubalis*) are a crucial source of red meat, milk, and milk products in most of the developing countries including Egypt [1]. The newborn calves of these species have poor immune system, which makes them susceptible to viral and bacterial infections. Diarrhea caused by *Escherichia coli* in newborn calves is one of the most economically important problems. *E*. *coli* is a gram-negative, rod-shaped, non-sporulating, flagellated, and facultatively anaerobic bacterium of the family *Enterobacteriaceae*. There are several diarrheagenic *E*. *coli* pathotypes including enteropathogenic *E*. *coli* (EPEC), Shiga-toxin producing *E*. *coli* (STEC), enterotoxigenic *E*. *coli* (ETEC) and enterohaemorrhagic *E*. *coli* (EHEC) [2, 3]. The most predominant pathotype in developed countries is ETEC [4], which has two groups of virulence factors e.g., enterotoxins and colonization factors (mainly fimbriae). The enterotoxins are divided into heat-stable (ST) and heat-labile enterotoxin (LT) [5]. The fimbriae serve as colonization factors and help the bacteria to adhere to the ileum. Many types of fimbriae have been identified but the most common in calves are F5 and F4 [6].

Several serotypes (e.g., O26:H11, O113:H21, O26, and O111) of STEC are zoonotic in nature [7]. STEC produces two types of toxins, Stx1 and Stx2, which were previously known as Shiga-like toxin or Verotoxin because of their similarity to the Shiga toxin produced by *Shigella*. *dystenteriae* and *S*. *sonnei*. The Stx1 and Stx2 can inhibit protein synthesis and produce apoptosis in target cells although Stx1 produces 10-fold more powerful cytotoxic effect in Vero cells than does Stx2 [8].

The EPEC does not produce enterotoxin or Shiga toxin [9] but its Eae gene encodes a protein (namely intimin) that has the ability to form attaching-effacing (A/E) lesions on intestinal cells. The necrotoxigenic *E*. *coli* (NTEC) produces a cytolethal distending toxin (Cdt) and two cytotoxic necrotizing factors CNF1 and CNF2. The CNF1 and CNF2 induce multi-nucleation and necrosis of eukaryotic cells [10]. Oral administration of NTEC in neonatal animals causes septicemia and enteritis [11]. The Cdt, first detected by Johnson and Lior in 1988 [12], is encoded by three genes known as CdtA, CdtB, and CdtC. It can impair host defense by holding cell cycle and by apoptosis in epithelial cells and lymphocytes and subsequent impairing of acquired immunity. It can also alter macrophage function leading to a pro-inflammatory response [13]. Molecular identification of virulence factors helps in *E*. *coli* classification, which can give an idea about the extent of benefit from the application of vaccines, which are not available against all types. In addition, identifying virulence factors can help predict the prognosis depending on the pathogenicity of each type that differs significantly according to the integrated virulence factors. Rate of change in virulence factors can serve as an alert about the endemic state of the pathogen with subsequent recommendation for trade limitation from the affected locality. The EPEC, STEC, and NTEC are opportunistic pathogens, which have also been isolated from non-diarrheic calves [10, 14, 15]. Understanding risk factors, e.g., farm size, age, sex, season, concurrent disease, colostrum management, calving factors, perinatal treatments, housing, feeding, genetics, and environment is very important in controlling *E*. *coli* infections and improving the health and performance of calves [16, 17, 18]. Currently, the control of infection in animals depends mainly on antibiotic treatment, which may lead to increased resistance among pathogenic and commensal bacteria [19]. Multidrug-drug resistant microorganisms from animals may contaminate the environment and find a way to infect humans [20]. Regular screening of antibiotic resistance in animals and their environment is helpful in identifying the extent of phenotypic variation among bacterial isolates and the depth of the problem. The aim of this study was to determine the pathotypes of *E*. *coli* in diarrheic calves of cattle and water buffaloes in Egypt and to determine their virulence factors and antibiotic susceptibility.

## Materials and methods

### Ethics statement

The rectal swabs were obtained from animals without anesthesia or pain relief by certified and well-trained veterinarians in the Faculty of Veterinary Medicine Clinic, Zagazig University, Egypt (Governmental Veterinary Clinic) following standard protocols. The swabs were sent to the laboratory for diagnosis after obtaining consent from the owners. The research presented in this manuscript was performed after receiving samples by the laboratories.

### Source of samples and history

Rectal swabs were obtained from animals reared in different places in Sharkia province, Egypt. These animals were brought to the Veterinary Clinic in the Faculty of Veterinary Medicine, Zagazig University, Sharkia, Egypt. The animals belonged to three age groups e.g., below one month (n = 80), between one and four months (n = 43) and four to twelve months (n = 27). The number of rectal swabs collected from diarrheic calves of cattle and buffaloes was 118 and 32, respectively. The samples were from 63 males and 87 females. A total of 49 and 101 samples were collected in spring (March- June) and winter (December-March), respectively. All samples were collected prior to beginning antibiotic treatment. The swabs were placed directly in nutrient broth and delivered to the microbiology laboratory.

### Bacterial and parasitological examination

For bacteriological examination, samples were streaked on MacConkey agar (Merck, Germany) followed by incubation at 37°C for 24 h to differentiate between lactose and non-lactose fermenters. Single colony of lactose fermenters was picked, suspended in sterile distilled water, and then streaked on eosin methylene blue agar (Merck, Germany) followed by incubation at 37°C. Non-lactose fermenters were streaked on XLD (xylose-lysine-deoxycholate agar). Standard biochemical tests (indole, methyl red, and citrate utilization) and growth on triple sugar iron (TSI) agar and motility test were also used to identify bacteria [21]. Confirmed *E*. *coli* colonies were picked and inoculated in tryptic soy broth. Following growth, glycerol was added and the stock cultures were stored at -20° C until used. For parasitological examination, samples were subjected to simple floatation technique to detect oocysts and nematode eggs [22]. No viral testing was carried out.

### Antimicrobial susceptibility testing

All isolates were tested against 14 antimicrobial agents belonging to eight different classes by disk diffusion method as recommended by the Clinical and Laboratory Standards Institute (CLSI) [23]. The antimicrobial agents tested were: ampicillin = AM (10 μg), amoxicillin-clavulanic acid = AMC (20/10μg, respectively), streptomycin = S (10 μg), kanamycin = K (30 μg), gentamicin = GM (10 μg), tetracycline = TE (30 μg), trimethoprim-sulfamethoxazole = SXT (1.25μg/23.75 μg respectively), nalidixic acid = NA (30 μg), ciprofloxacin = CIP (5 μg), imipenem = IPM (10 μg), ceftazidime = CAZ (30μg), cefotaxime = CTX (30 μg), ceftriaxone = CRO (30 μg), and aztreonam = ATM (30 μg). The inoculum was standardized to 0.5 McFarland unit using a nephelometer. The antibiotic discs were placed on the surface of the agar using a disc dispenser and the plates were incubated at 37°C for 18 hours after which the zones of inhibition were measured. Interpretation of results was performed according to CLSI recommendations. Isolates with intermediate resistance were considered as resistant and were added to the resistant count.

### Virulence factors

Overnight cultures of bacteria in 2 ml of trypticase soy broth were centrifuged to pellet bacteria. After discarding the supernatant, the pellet was washed twice in distilled water followed by vortexing and centrifugation at 12,000*xg* for 5 min. For DNA extraction, 300μL of 10% chelex 100 resin (Bio-Rad, USA) was added and the pellet was re-suspended by vortexing. The tubes were incubated at 99° C for 30 min followed by vortexing and centrifugation at 12,000 *xg* for 10 min. The DNA-containing supernatant was collected and stored at -20°C. The primers and PCR conditions are shown in Table 1. Briefly, incubation at 95°C for 10 min was used as an initial denaturation step followed by 35 cycles of amplification. Each cycle consisted of a denaturation step at 95°C for 1 min followed by annealing for 1 min at different temperatures according to the target gene (Table 1) and elongation at 72°C for 1 min. Final elongation step was done at 72°C for 10 min.

**Table 1:** Primers used for detection of virulence factors.

Primer^A^	Sequence (5’-3’)	Tn	Amplicon size	Reference
Hly-F	GAGCGAGCTAAGCAGCTTG	56°C	889	[9]
-R	CCTGCTCCAGAATAAACCACA			
Stx1-F	CAGTTAATGTGGTGGCGAAGG	56°C	348	[9]
-R	CACCAGACAATGTAACCGCTG			
Stx2-F	ATCCTATTCCCGGGAGTTTACG	56°C	584	[9]
-R	GCGTCATCGTATACACAGGAGC			
Eae-F	TGCGGCACAACAGGCGGCGA	64°C	629	[9]
-R	CGGTCGCCGCACCAGGATTC			
CNF1-F	GGGGGAAGTACAGAAGAATTA	56°C	1111	[24]
-R	TTGCCGTCCACTCTCTCACCAGT			
CNF2-F	TATCATACGGCAGGAGGAAGCACC	56°C	1240	[24]
-R	GTCACAATAGACAATAATTTTCCG			
Cdt3-F	GAAAATAAATGGAATATAAATGTCCG	58°C	555	[24]
-R	TTTGTGTCGGTGCAGCAGGGAAAA			
LT-F	GCACACGGAGCTCCTCAGTC	56°C	218	[9]
-R	TCCTTCATCCTTTCAATGGCTTT			
ST-F	AGGAACGTACATCATTGCCC	53°C	521	[9]
-R	CAAAGCATGCTCCAGCACTA			
F5-F	TATTATCTTAGGTGGTATGG	44°C	314	[6]
-R	GGTATCCTTTAGCAGCAGTATTTC			

^A^ = Hly = hemolysin gene, Stx1 = Shiga toxin 1, Stx2 = Shiga toxin 2, Eae = attaching-effacing gene, CNF1 = Cytotoxic necrotizing factor 1, CNF2 = Cytotoxic necrotizing factor 2, Cdt3 = Cytolethal distending toxin 3, LT = Heat labile enterotoxin; ST = heat stable enterotoxin, F5 = Fimbria

### Statistical analysis

The evaluation of the potential risk factors associated with *E*. *coli* pathotypes was performed as follows: descriptive statistics on the frequency of age, species, gender, and season of sampling were calculated; the total number of samples (n = 150) was reduced to 96 samples after excluding observations with missing values in any variable and outliers, using the 1.5*IQR rule. Since the sample size of this study was modest, no variable selection was done. Logistic regression models (one per *E*. *coli* pathotype) were used to assess the association between the isolation of each *E*. *coli* pathotype and age, species, sex, and season. Adjusted odds ratios (OR) of isolating *E*. *coli* pathotypes and their 95% confidence intervals are reported. OR’s with P values< 0.05 are considered statistically significant. All analyses were performed in Stata 15.1 (StataCorp LLC, TX, USA).

## Results

### Isolation and identification

Among the parasitic agents, *Cryptosporidium* and gastrointestinal nematodes were the highest (33.3% and 17.3%, respectively). Non-lactose fermenters, not including possible non-lactose fermenter *E*. *coli*, were low in number while lactose fermenter *E*. *coli* was found in 88 of 150. As many as 54 of 88 (61.4%) had mixed infection with other bacterial and/or parasitic agents (Table 2). The highest rate of mixed infection with *E*. *coli* was with *Cryptosporidium* (15.3%) and gastrointestinal nematodes (10%).

**Table 2:** Microorganisms and parasites detected in diarrheic calves.

Bacteria and parasites detected	Cattle (n = 118)				Buffaloes (n = 32)				Total No. (%) positive
	>1m	1–4 m	4-12m	Total	>1m	1–4 m	4–12 m	Total	
*Escherichia coli*	18	2	-	20	8	6	-	14	34 (22.6)
*E*. *coli* + *Enterobacter*	3	-	-	3	-	-	-	0	3 (2.0)
*E*. *coli* + *Klebsiella*	4	-	-	4	-	-	-	0	4 (2.6)
*E*. *coli* + Coccidia	2	4	3	9	-	-	-	0	9 (6.0)
*E*. *coli*+ *Cryptosporidium*	4	10	7	21	-	2	-	2	23 (15.3)
*E*. *coli* + Nematodes	2	9	-	11	-	2	2	4	15 (10.0)
*Enterobacter spp*.	3	7	3	13	2	1	1	4	17 (11.3)
*Citrobacter spp*.	1	2	-	3	1	3	1	5	8 (5.3)
*Klebsiella spp*.	2	4	2	8	-	-	-	0	8 (5.3)
*Salmonella spp*.	-	3	5	8	-	3	1	4	12 (8.0)
*Shigella spp*.	-	3	-	3	-	-	-	0	3 (2.0)
Coccidia	3	8	1	12	-	-	2	2	14 (9.3)
*Cryptosporidium*	4	30	7	41	1	5	3	9	50 (33.3)
Nematodes	1	2	16	19	-	3	4	7	26 (17.3)

### Virulence profile

**Fig 1:**
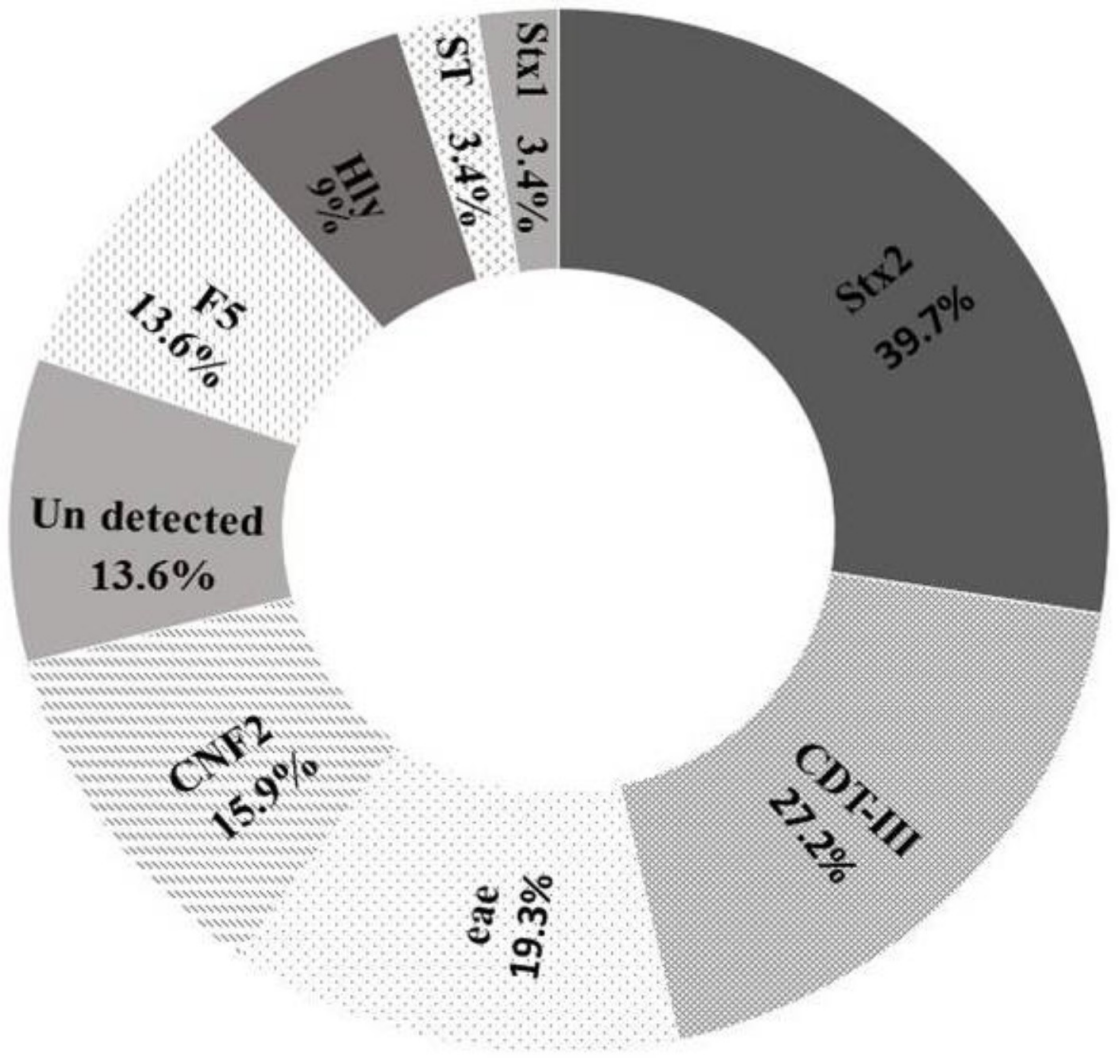
Percentage of different virulence genes among the examined *E*. *coli* isolates.

The virulence profile and percent distribution of various genes in *E*. *coli* isolates are shown in Table 3 and Fig 1. The distribution of different *E*. *coli* genotypes per age group is given in S1 Appendix. EPEC was detected in 4.5% samples; the Eae gene was present in 17 (19.3%) isolates. The STEC were present in 31 of 88 (35.2%) isolates. The Stx1gene was present in only three isolates while Stx2 was the most predominant gene among all genes examined (39.7%). The ETEC represented 15.9% of the isolates. None of these isolates was positive for LT toxin gene while three isolates were positive for ST gene. F5 gene was present in 11 of 14 ETEC isolates. NTEC was in 30.6% of the isolates. The most predominant genes among NTEC strains were Cdt3 and CNF2 (in 24 and 14 isolates, respectively). CNF1 was not detected in any of the samples. None of the selected genes was detected in 12 of 88 isolates.

**Table 3:** Virulence gene profile of *E*. *coli* isolates.

Pathotype	Virulence genes	No of isolates
EPEC (n = 4)	Eae	4
STEC (n = 31)	Stx2	16
	Stx2; Eae	7
	Stx2; Hly	2
	Stx1; Eae	3
	Eae, Hly	1
	Stx2; Eae, Hly	2
ETEC (n = 14)	LT	0
	ST	3
	F5	11
NTEC (n = 27)	Cdt3	7
	Cdt3; Stx2	4
	CNF1	0
	CNF2	1
	CNF2; Stx2	2
	Cdt3; CNF2	9
	Cdt3; CNF2; Stx2	1
	Cdt3; Hly	2
	Cdt3; CNF2; Stx2; Hly	1
None detected	No genes	12

### Risk factors

The odds of isolating ETEC from male calves was 3.6 times (95% CI: 1.1, 12.4; P value = 0.042) that of female calves after adjusting for age, species, and season. The odds of isolating NTEC in male calves was72.9% lower (95% CI: 91.3% lower, 15.7% lower; P value = 0.024) than that in female calves. In contrast, the odds of isolating STEC in winter was 3.3 times (95% CI: 1.1, 10.3; P value = 0.037) that in spring. Table 4 shows a complete list of odds ratios (95% confidence intervals and P values) by variable and *E*. *coli* pathotype.

**Table 4:** Association of potential risk factors and *E*. *coli* pathotypes in calves.

Pathotype	Potential risk factors (95% CI)			
	Age	Species	Sex	Season
EPEC	1.01(0.96, 1.07)	0.71(0.07, 7.3)	1.66(0.22,12.36)	N/A
ETEC	0.97(0.93, 1.02)	0.52(0.12, 2.24)	3.61(1.05, 12.41) *	1(0.24, 4.16)
NTEC	1.01(0.98, 1.04)	0.98(0.26, 3.68)	0.27(0.09, 0.84) *	2.39(0.76, 7.48)
STEC	1.02(0.99, 1.05)	0.48(0.14, 1.63)	1.24(0.49, 3.15)	3.32(1.07, 10.29) *

Values are odds ratios adjusted for other variables in the model at 95% CI: 95% confidence interval. The reference groups for species, sex, and season were buffalo, female, and spring, respectively.

(N/A) = all EPEC were isolated in winter; therefore, season could not be evaluated in that model.

(*) P value <0.05.

### Antibiograms

**Fig 2:**
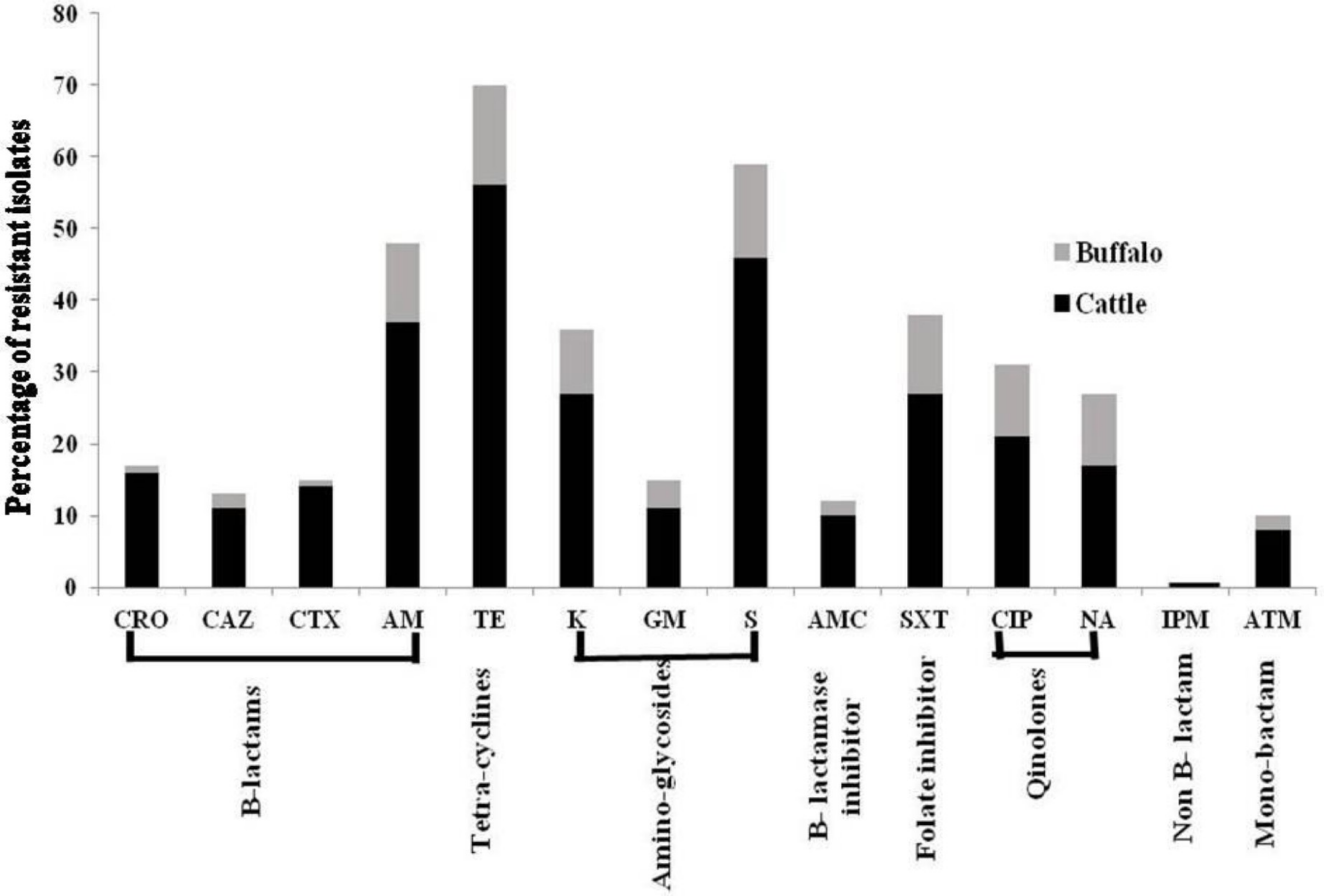
Diagram showing the rate of antimicrobial resistance among diarrheic cattle and buffalo calves against 14 different antimicrobial agents.

**Fig 3:**
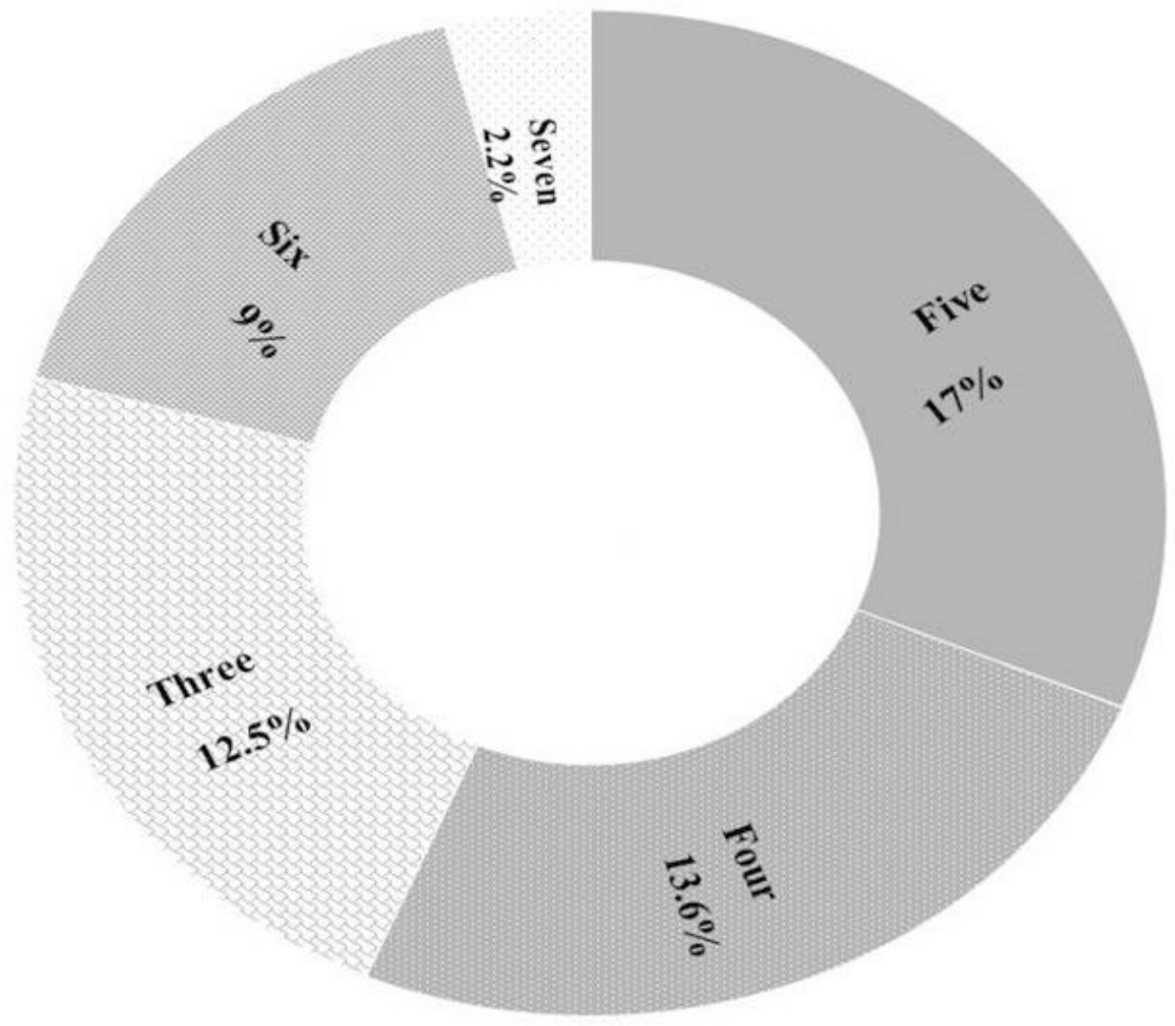
Percentage of different MDR groups; Alphabetic numbers in the pie chart refer to the number of ineffective drug groups, while percentages refer to the percent of resistant *E*. *coli* in each group.

Antibiograms for 88 *E*. *coli* isolates are shown in Table 5 and Fig 2. The highest resistance was detected against tetracycline (79.5%) and streptomycin (67.0%) followed by ampicillin (54.5%) and trimethoprim-sulfamethoxazole (43%). The lowest resistance was against ceftazidime (14.8%) and aztreonam (11.3%). None of the isolates was resistant to imipenem. Resistance to aminoglycoside and tetracycline groups was the highest while resistance to B- lactam and monobactam was the lowest. Multidrug resistance for drugs classes (against 3–7 antibiotics) was detected in 48 (54.5%) isolates (Fig 3).

**Table 5:** Antibiograms of *E*. *coli* isolates. The number in the table refers to the numbers of resistant plus intermediate isolates against each antimicrobial agents in cattle and buffalo.

Antibicrobial group		Β-lactams				Tetra-cyclines	Amino-glycosides			Β-lactamase inhibitor	Folate inhibitor	Quinlones		Non-β-lactam	Mono-bactam
Antimicrobial agents		CRO	CAZ	CTX	AM	TE	K	GM	S	AMC	SXT	CIP	NA	IPM	ATM
**Numbers of resistant isolates**	Cattle	15	11	14	37	56	27	11	46	10	27	21	17	0	8
	Buffalo	1	2	1	11	14	9	4	13	2	11	10	10	0	2
	Total	16	13	15	48	70	36	15	59	12	38	31	27	0	10
	Percent	18.2	14.8	17	54.5	79.5	41	17	67	13.6	43	35	30.7	0	11.3

AM = ampicillin, AMC = amoxicillin-clavulanic acid, S = streptomycin, K = kanamycin, GM = gentamicin, TE = tetracycline, SXT = trimethoprim-sulfamethoxazole, NA = nalidixic acid, CIP = ciprofloxacin, IPM = imipenem, CAZ = ceftazidime, CTX = cefotaxime, CRO = ceftriaxone, ATM = aztreona

## Discussion

Neonatal diarrhea is the main cause of economic loss in suckling calves from the first week to 3 months of age. *E*. *coli* is a major cause of neonatal diarrhea in both cattle and water buffaloes [25]. The acquisition of foreign genes located on mobile genetic structures such as transposons, phages, plasmids, and pathogenicity islands helps *E*. *coli* become more pathogenic and virulent. Concurrent infections with parasitic and bacterial pathogens can further complicate the problem.

The rate of isolation of *E*. *coli* (88 of 150 animals or 58.7%) in our study is similar to that reported in previous surveillance studies of diarrheic calves in Egypt [26, 27]. Surveillance data from developed countries, however, show a lower rate of *E*. *coli* infections, which is probably due to differences in rearing, sanitary conditions, and management practices [28, 29]. Relationship between various risk factors and passive immunity play an important role in disease prevention. In this study, samples from calves less than 60 days of age were included in the logistic regression models. In some cases, data on age, sex, season and locality were not available due to the absence of farm records. Complete investigation for other risk factors, especially colostrum management, calving factors, perinatal treatments, housing, feeding, genetics, and environmental factors is needed to reduce the incidence of *E*. *coli* infections. In this regard, National database records from questionnaire surveys should make future studies easier [30].

We used PCR for detection of virulence genes in *E*. *coli*. Previous studies have shown that PCR is 100% specific and 98% sensitive [24, 31]. Low percentage of ETEC in our study could be due to the availability of a commercial fimbrial vaccine, which is routinely given to pregnant cattle in Egypt. High antibody titers in colostrum supplies the calves with passive immunity during the critical early neonatal period in which the susceptibility to infection is high. The ETEC fimbriae are the most necessary part in adhesion and colonization of bacteria, and the neutralization of this protein stops bacterial pathogenesis [32]. In contrast, vaccination against other pathotypes is still a problem. For instance, STEC vaccine can suppress the antigen-specific cellular immune response in cattle with continuous shedding of microorganism [33].

None of the 11 isolates that were positive for F5 had enterotoxin genes a finding similar to that reported by Nguyen et al. [34]. ETEC without fimbrial genes does not have the ability to colonize the small intestine and hence are nonpathogenic. Our results showed that three ETEC isolates had ST without F5 fimbriae. In contrast to Borriello et al. [24], no Lt toxin was detected. Strains carrying Eae but not Stx1 and Stx2 variants, are considered as potential EPEC while strains carrying Eae with Stx1 or Stx2 variants are considered as STEC [35]. Stx2 has a powerful cytotoxic effect on endothelial cells and is associated with serious infections [36, 37]. The high Stx2 percentage is associated with clinical signs, which agrees with Wani et al. [38], who stated that the Stx2 gene was more prevalent than Stx1 and that both were associated with Eae gene in STEC strains. This finding, however, is in contrast to an earlier report that relates signs with the presence of Stx1 with Eae genes [39]. Eae genes were detected in 19.3% of *E*. *coli* isolates, which is in agreement with Ishii et al. [35] but is in contrast to Chandran and Mazumder [40] and Caprioli [37]. Presence of Stx2 among NTEC as NT2 and Cdt3 may indicate the emergence of a new pathotype.

The absence of CNF1 in the tested samples indicates that it plays a limited role in disease pathogenesis [41] although it has been recorded in many diarrhea outbreaks [9]. Many virulence factors are associated with the hemolysin activity, but we focused only on the evaluation of the most common virulence factors, *hlyA*, associated with hemolysin activity in *E*. *coli*. Some other genes, such as *sheA* and *clyA* were not identified, which might have indicated the possibility of increased hemolysis activity in phenotypic investigations. Detection of HlyA with CNF may be because of a chromosomal linkage in pathogenicity islands (PAIs), which carry a virulence-associated gene coding for CNF, HlyA [42]. Although CNF1 was detected in some diarrheic calves, its prevalence is uncommon among *E*. *coli* causing diarrhea [9, 24] and it is more common among extra-intestinal *E*. *coli* infections [31].

The odds of isolating ETEC from male calves was greater than female calves probably because the owners tend to provide better care for female calves; the latter are considered as the main unit for production, especially in dairy herds. It is also known that physical activity and vitality are higher in male calves, which may further increase their chances of exposure to infectious agents [43]. The opportunistic nature of NTEC may be the cause for an increased rate in the female. In Egypt, female calves are more exposed to the stress of early weaning and transportation into new barns for following certain nutritional programs or may relate to the stress of hormonal changes [44]. The increasing rate of STEC in winter, rainy cold season, may relate to cold stress or stress of ration change from dry or semi-dry food to green food [45].

Antibiograms are considered more reliable for the detection of antibiotic resistance than genotypic resistance gene detection [46]. The main concern with the presence of drug resistance in *E*. *coli* is that these resistance genes can be propagated and transferred not only to other bacteria but also to other hosts including humans. Thus, drug resistance can be transferred to bacteria that have never been exposed to the drug if excessive amounts of antimicrobials are used in the prophylaxis and treatment of agricultural animals and people [47, 48, 49]. The modified bacteria with the integrated resistance genes can be infectious for new hosts including humans. High resistance to tetracyclines and streptomycin (penicillin-streptomycin mix) in our study was high, possibly because of the use of these broad-spectrum antibiotics by paramedical personnel and farmers. None of the isolates was resistant to imipenem, which is not surprising because there is no trade medicine for veterinary use that contains imipenem as an active ingredient in Egypt [50]. Resistance to trimethoprim-sulfamethoxazole was high possibly because Egyptian paramedical people use this drug as a choice for any gut infection.

Although there are no veterinary trade drugs that contain aztreonam, nalidixic acid or ceftazidime as active ingredients [50], resistance was detected against them. This may be due to: (i) the usage of human drugs for treatment of infected animals; (ii) transfer of human bacteria, in which resistance has already developed, to animal hosts [47]; and (iii) excretion of these antibiotics and their metabolites in human sewage and sludge, from which resistance is subsequently transferred to animals following the use of sludge as a fertilizer or through irrigation with waste water [47, 51]. In conclusion, identification of virulence and risk factors and the antibiograms of *E*. *coli* should inform veterinarians about the endemicity, pathogenicity and possible prognosis of *E*. *coli* infections in calves thereby reducing the indiscriminate usage of antibiotics.

## Supporting information

S1 Appendix Distribution of different *E*. *coli* genotypes according to age groups. (DOCX)
