# Supplementary material for: Virulence factors and antibiograms of Escherichia coli isolated from diarrheic calves of Egyptian cattle and water buffaloes
Source: PLoS One. 2020 May 11;15(5):e0232890. doi: 10.1371/journal.pone.0232890 (PMC7213691; doi:10.1371/journal.pone.0232890)
Supplement: S1 Appendix — (DOCX) [file pone.0232890.s001.docx]

S1 Appendix. Distribution of different *E. coli* genotypes according to age groups.

| **Genotypes** | **Buffaloes** | | | | **Cattle** | | | |
| --- | --- | --- | --- | --- | --- | --- | --- | --- |
|  | **>1m** | **1-4 m** | **4-12m** | **Total** | **>1m** | **1-4 m** | **4-12m** | **Total** |
| **EPEC** | - | 1 | - | 1 | 2 | 1 | - | 3 |
| **STEC** | 6 | 3 | - | 9 | 9 | 15 | - | 24 |
| **ETEC** | 2 | 1 | - | 3 | 9 | 1 | - | 10 |
| **NTEC** | - | 3 | - | 3 | 13 | 8 | 2 | 23 |
